# Association of intensive care unit or paediatric intensive care unit admissions with the method of transporting patients: a multicentre retrospective study

**DOI:** 10.1186/s12873-022-00710-9

**Published:** 2022-09-07

**Authors:** Tadashi Ishihara, Ken Okamoto, Hiroshi Tanaka

**Affiliations:** Department of Emergency and Critical Care Medicine, Juntendo University, Urayasu Hospital, 279-0021, 2-1-1, Tomioka, Urayasu-city, Chiba Japan

**Keywords:** Transport, Paediatric intensive care, Retrospective study, Epidemiology

## Abstract

**Background:**

Reports regarding transportation methods of severely critical patients admitted to an intensive care unit (ICU) or paediatric ICU (PICU) are limited. In an attempt to address this research gap, this study aimed to test the hypothesis that prognosis is worse in patients transported by family members.

**Methods:**

This multicentre study collected data from the Japanese Registry of Paediatric Acute Care database. Data concerning patients aged ≤16 years admitted to a participating hospital ICU or PICU and their transportation method to the hospital were extracted and divided into two groups: transported by family and transported by emergency medical services (EMS).

**Results:**

Of the 2963 patients who met the criteria, 871 (29.4%) were transported by family and 2092 (70.6%) were transported by EMS. Significantly more patients with chronic conditions (551 patients, 63.3% vs. 845 patients, 40.4%; *p* <  0.01) or respiratory failure (414 patients, 47.5% vs. 455 patients, 21.7%; *p* <  0.01) were admitted to the ICU or PICU in the family transport group. There was no significant difference in survival rate between EMS and family transport group, matched by PIM2, chronic condition status and transport distance (OR:1.17, 95%CI:0.39–3.47, *p* = 0.78).

**Conclusion:**

The results of this study show that the transportation method does not affect the survival rate of paediatric patients. The proportion of patients with chronic conditions or those admitted because of respiratory failure was higher in the family transport group than in the EMS group. Therefore, as these patients are more likely to be admitted to the ICU or PICU, it is important to provide prompt respiratory care and medical interventions to achieve the best outcomes.

## Background

Some studies have reported various admission routes to the intensive care units (ICUs) or paediatric ICUs (PICUs) [1–3]. The PICU has multiple sources of admissions, such as from surgical and emergency departments and medical units and transfers from other hospitals when more intensive care is required. Some paediatric studies have reported that 20–68% of PICU cases were admitted from the emergency department [1, 3, 4]. Moreover, some studies have reported higher ICU-related mortality in patients transferred to the ICU from other wards within the same hospital than in patients admitted from other sources [3, 5–7]. The admission source was an important factor associated with fatal outcomes in the PICU of a tertiary hospital; the mortality rate in patients admitted to the ICU or PICU from the general ward due to deterioration in condition was two times higher [2] [8], El Halal et al. reported that paediatric mortality was doubled in ICU patients admitted from general wards compared with those admitted from the paediatric emergency department [2].

Generally, patients with severely critical conditions are transferred to the hospital by emergency medical services (EMS); however, few studies have reported the transportation method to the hospital of patients admitted to the ICUs or PICUs. The EMS system in Japan is very characteristic. EMS can be used free of charge, even if the patient does not have any insurance. The EMS system is maintained in each area, and people can use the system almost everywhere in Japan. The procedures that the EMS staff can perform are very limited in Japan, especially for paediatric patients. They can only provide oxygen, ventilatory support with bag-bulb mask, or chest compression.

Data on transportation methods to the hospital of paediatric patients admitted to the PICUs may help identify appropriate resources related to intensive care utilisation because of the limited PICU beds in Japan. Japan only has 23 hospitals with PICUs, so in areas without a PICU, critically ill paediatric patients are admitted to ICUs containing both adult and paediatric patients.

It is essential to understand variations in the method of hospital admissions and related patterns to predict patients’ outcomes, especially those admitted to ICUs or PICUs. Thus, we sought to describe the transportation patterns of patients admitted to the ICUs or PICUs in Japan. This study aimed to test the hypothesis that prognosis is worse in patients transported by family members. It is also important to clarify the characteristics of patients admitted to ICUs or PICUs to provide early medical interventions and prevent deterioration.

## Methods

### Dataset

The Japanese Registry of Pediatric Acute Care (JaRPAC) is a multicentre clinical database of ICU and PICU patients founded by the Japanese Society for Emergency Medicine. It was initiated in April 2014, with the aim of evaluating critically ill paediatric patients and reducing their mortality rate. The JaRPAC database contains anonymised information regarding patient demographics, admissions, treatment, and outcomes, as well as scoring systems for severity and mortality [9]. Paediatric patients aged ≤16 years admitted in ICUs or PICUs are eligible for inclusion in this registry, and data are available on a per capita basis. The data are collected from admission until discharge from the ICU or PICU. The National Center for Child Health and Development is the primary institute managing this registry data, and hospitals affiliated with this institute were selected to participate in the registry. This includes 12 PICUs in children’s hospitals and 11 ICUs at critical care centres.

**Fig. 1:**
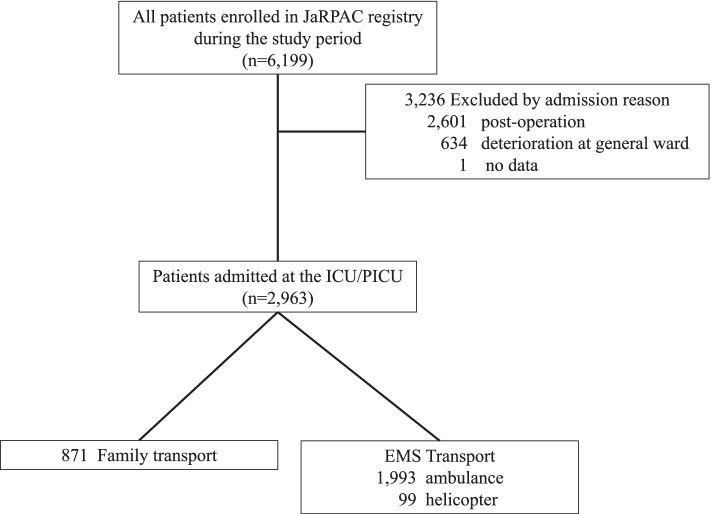
Inclusion criteria of the study. JaRPAC: Japanese Registry of Pediatric Acute Care, ICU: intensive care unit, PICU: paediatric intensive care unit, EMS: emergency medical service

Patients aged ≤16 years consecutively admitted to the ICU or PICU in a participating hospital between April 2014 (when the JaRPAC was started) and March 2017 were included in this study. This study was initiated to investigate patterns among patients admitted to the ICU or PICU from the emergency department; therefore, patients admitted to the ICU or PICU by postoperative management or deterioration in the general ward were excluded from the study (Fig. 1).

### Ethical information

This study was approved by the Institutional Review Board of Juntendo University Urayasu Hospital, Chiba, Japan (30–025) and was conducted in accordance with the principles outlined in the 1964 Declaration of Helsinki and its later amendments. The need for informed consent was waived by the Institutional Review Board owing to the retrospective nature of the study. We followed the STROBE reporting guidelines while conducting this study.

### Study design

This multicentre retrospective study was based on the data collected from JaRPAC. Data concerning the method of transport to the hospital were extracted from the database and divided into two groups, namely, transported by family members and by EMS. All patients admitted to the PICU or ICU first came through the emergency department. The cause of admission to the ICU or PICU was registered and classified into five categories: respiratory failure (requiring oxygenation or ventilatory support), circulatory failure (requiring circulatory support), neurological dysfunction, observation, and treatment for post-cardiopulmonary resuscitation. The final diagnosis of the patients in the case of intrinsic disease was coded based on the International Classification of Diseases v. 10 and categorised into one of nine groups to ensure sufficient patient numbers for analysis as follows: cardiovascular, respiratory, neuromuscular, congenital/genetic, gastrointestinal/hepatobiliary-pancreatic, hematologic/oncologic, renal, sepsis, and metabolic/endocrinologic groups [10, 11]. Extrinsic causes were categorised as trauma, asphyxia, poisoning, burns, drowning, suicide, or heatstroke. Chronic conditions were defined according to Feudtner et al.’s definition, which states that a chronic condition involves either several organ systems or one organ system severely enough to require speciality paediatric care and probably some period of hospitalisation in a tertiary care centre [12].

The Paediatric Index of Mortality 2 (PIM2) was used to measure disease severity in patients. The PIM2 score is calculated from various coefficients determined by Slater et al. [9]. The values used to calculate PIM2 resulted from the first face-to-face contact between patients and physicians at ICUs or PICUs. Data for some factors were not obtained for all cases; these factors were not included in the PIM2 calculations in these cases. Patient survival was defined as discharge from the ICU or PICU.

The duration of interventions performed in the ICU or PICU was compared between the groups. Interventions included invasive mechanical ventilation (IMV), non-invasive positive pressure ventilation continuous haemodiafiltration (CHDF), plasma exchange, polymyxin B-immobilised fibre column-direct haemoperfusion, extracorporeal membrane oxygenation (ECMO), intracranial cerebral pressure sensor placement, central venous access catheterisation (CV), peripherally inserted central catheterisation (PICC), and arterial line catheterisation (A-line).

### Statistical analyses

Data regarding age, length of ICU or PICU stay, PIM2, and duration of interventions from JaRPAC were skewed, and medians with interquartile ranges were used for numerical variables. Numerical variable differences between the two groups were compared using the Mann-Whitney *U* test. The chi-square test was used to compare sex distribution as well as mortality, time of admission, reasons for admission, and final diagnosis. Data regarding mortality, transport distance and chronic condition status were analyzed by age category and PIM2 risk intervals. To assess the independent effect of transportation type, multivariable logistic regression analysis of survival was performed. PIM2, chronic condition status and transport distance were included as variables of multivariable logistic analysis. Data management and statistical analyses were performed using EZR software (Y Kaneda, Saitama Medical Center, Jichi Medical University, Saitama, Japan). A *p* value < 0.01 was considered significant.

## Results

A total of 23 hospitals contributed data that were used in the study, and 6199 paediatric patients were registered with JaRPAC during the study period. Of these patients, 2963 met the study criteria, among whom 871 were brought to the hospital by their family (family transport group) and 2092 by the EMS (EMS group) (Fig. 1). The patients’ characteristics are shown in Table 1. Among these, 9 (1.0%) patients in the family transport group and 80 (3.8%) in the EMS group died (*p* <  0.01). The median mortalities predicted by PIM2 scores were 1.1% (0.8–2.2) and 1.5% (1.0–5.2) for the family transport and EMS groups, respectively (*p* <  0.01). A significant difference was found in the number of patients with chronic conditions between the two groups (*p* <  0.01).

**Table 1:** Patient’s characteristics

Characteristics	Family transport group (*n* = 871)	EMS transport group (*n* = 2092)	*p*-value	OR
Age (months), median (IQR)	28 (8–81)	28 (8–81)	0.328	–
Gender (male, %)	460 (52.8)	1143 (54.6)	0.374	0.93
Length of ICU/PICU stay (days), median (IQR)	4 (2–6)	4 (2–8)	0.137	–
PIM2 (%), median (IQR)	1.1 (0.8–2.2)	1.5 (1.0–5.2)	< 0.01^*^	–
Chronic condition (%)	551 (63.3)	845 (40.4)	< 0.01^*^	2.54
Mortality (%)	9 (1.0)	80 (3.8)	< 0.01^*^	0.27
Distance to hospital	5.3 (3.1–9.6)	9.8 (4.9–17.4)	< 0.01^*^	–

*OR* Odds ratio, *EMS* Emergency medical service, *IQR* Interquartile range, *ICU* Intensive care unit, *PICU* Paediatric intensive care unit, *PIM2* Paediatric index of mortality 2

* *p* <  0.01

Table 2 shows the causes of ICU and PICU admissions. A significantly higher number of patients were admitted to the ICU or PICU for respiratory failure (414 patients, 47.5% vs. 455 patients, 21.7%; *p* <  0.01) or circulatory failure (132 patients, 15.2% vs. 179 patients, 8.6%; *p* <  0.01) in the family transport group than in the EMS group. By contrast, significantly more patients were admitted to the ICU or PICU for neurological dysfunction in the EMS group than in the family transport group (783 patients, 37.4% vs. 151 patients, 17.3%; *p* <  0.01).

**Table 2:** Admission reasons to ICU/PICU

Admission reasons	Family transport group (*n* = 871)	EMS transport group (*n* = 2092)	*p*-value	OR
Respiratory failure (%)	414 (47.5)	455 (21.7)	< 0.01^*^	3.26
Circulatory failure (%)	132 (15.2)	179 (8.6)	< 0.01^*^	1.91
Neurological dysfunction (%)	151 (17.3)	783 (37.4)	< 0.01^*^	0.35
Observation (%)	172 (19.7)	578 (27.6)	< 0.01^*^	0.65
Treatment for post-CPA resuscitation (%)	2 (0.2)	97 (4.6)	< 0.01^*^	0.05

*OR* Odds ratio, *ICU* Intensive care unit, *PICU* Paediatric intensive care unit, *EMS* Emergency medical service, *CPA* Cardiopulmonary arrest

*p <  0.01

Table 3 shows the categories of the final diagnoses of patients admitted to ICUs and PICUs. The occurrence of intrinsic disease was significantly higher in the family transport group than in the EMS group (805 patients, 92.4% vs. 1603 patients, 76.6%; *p* <  0.01). Among the intrinsic group, respiratory disease was the leading diagnosis in the family transport group; its occurrence was significantly higher than that in the EMS group (411 patients, 51.1% vs. 415 patients, 25.9%; *p* <  0.01). By contrast, neuromuscular disease was the leading diagnosis in the EMS group; its occurrence was significantly higher in the EMS group than in the family transport group (672 patients, 41.9% vs. 138 patients, 17.1%; *p* <  0.01).

**Table 3:** Categories of final diagnosis at ICU/PICU

Diagnosis	Family transport group (*n* = 871)	EMS transport group (*n* = 2092)	*p*-value	OR
Intrinsic disease	805 (92.4)	1603 (76.6)	< 0.01^*^	3.72
Neuromuscular disease (%)	138 (17.1)	672 (41.9)	< 0.01^*^	0.29
Respiratory disease (%)	411 (51.1)	415 (25.9)	< 0.01^*^	2.99
Cardiovascular disease (%)	60 (7.5)	114 (7.1)	0.802	1.05
Gastrointestinal, Hepato-Biliary-Pancreatic disease (%)	66 (8.2)	148 (9.2)	0.405	0.88
Renal disease (%)	17 (2.1)	36 (2.2)	0.884	0.94
Infectious disease (%)	63 (7.8)	61 (3.8)	< 0.01^*^	2.15
Oncologic disease (%)	12 (1.5)	32 (2.0)	0.424	0.74
Metabolic/Endocrinologic disease (%)	15 (1.9)	46 (2.9)	0.169	0.64
Immunology disease	11 (1.4)	33 (2.1)	0.262	0.66
Other (%)	12 (1.5)	46 (2.9)	0.047	0.51
Extrinsic disease	66 (7.6)	489 (23.4)	< 0.01^*^	0.27
Trauma (%)	50 (75.8)	373 (76.3)	0.879	0.97
ISS > 15 (%)	17 (34)	140 (37.5)	0.755	0.86
Foreign body (%)	8 (12.1)	26 (5.3)	0.049	2.46
Poisoning (%)	5 (7.6)	18 (3.7)	0.176	2.14
Burn (%)	3 (4.5)	25 (5.1)	1	0.88
Suicide (%)	0	10 (2.0)	0.617	0
Drowning (%)	0	34 (7.0)	0.024	0
Heatstroke (%)	0	3 (0.6)	1	0

*OR* Odds ratio, *ICU* Intensive care unit, *PICU* Paediatric intensive care unit, *EMS* Emergency medical service

*p <  0.01

Table 4 lists the therapies applied to and devices used for the patients. A significantly higher number of patients in the EMS group received IMV (910 patients, 43.5% vs. 219 patients, 25.1%; *p* <  0.01), CHDF (95 patients, 4.5% vs. 10 patients, 1.1%; *p* <  0.01), ECMO (26 patients, 1.2% vs. 0 patient, 0%; *p* <  0.01), intracranial pressure sensor placement (31 patients, 1.5% vs. 1 patient, 0.1%; *p* <  0.01), CV line placement (583 patients, 27.9% vs. 128 patients, 14.7%; *p* <  0.01), A-line placement (976 patients, 46.7% vs. 281 patients, 32.3%; *p* <  0.01), and PICC line placement (292 patients, 14.0% vs. 70 patients, 8.0%; *p* <  0.01). On the contrary, the number of patients with non-invasive mechanical ventilation was larger in the family transport group than in the EMS group (14 patients, 13.1% vs. 160 patients, 7.6%; *p* <  0.01). Additionally, the placement duration of A-line (5 days vs. 4 days; *p* <  0.01) and PICC line (6 days vs. 4 days; *p* <  0.01) in the EMS group was significantly longer than that in the family transport group.

**Table 4:** Procedures at ICU/PICU

Procedures	Family transport group (*n* = 871)	EMS transport group (*n* = 2092)	*p*-values	OR
IMV (%) days, median (IQR)	219 (25.1) 4 (2–7)	910 (43.5) 5 (2–8)	< 0.01^*^ 0.08	0.43
NPPV (%) days, median (IQR)	114 (13.1) 2 (2–4)	160 (7.6) 3 (2–4)	< 0.01^*^ 0.74	1.82
CHDF (%) days, median (IQR)	10 (1.1) 4 (2–6.5)	95 (4.5) 5 (3–8.5)	< 0.01^*^ 0.285	0.24
PEX (%) days, median (IQR)	7 (0.8) 3 (1–3)	49 (2.3) 4 (3–5)	< 0.01^*^ 0.026	0.34
PMX-DHP (%) days, median (IQR)	3 (0.3) 3 (3–3)	7 (0.3) 2 (1–2.5)	1 0.08	1.03
ECMO (%) days, median (IQR)	0	26 (1.2) 5 (3–7.75)	< 0.01^*^	0
ICP sensor (%) days, median (IQR)	1 (0.1) 6 (6–6)	31 (1.5) 6 (3–7)	< 0.01^*^ 0.913	0.08
CVC (%) days, median (IQR)	128 (14.7) 5 (3–8)	583 (27.9) 6 (4–8)	< 0.01^*^ 0.0988	0.45
A-line (%) days, median (IQR)	281 (32.3) 4 (2–6)	976 (46.7) 5 (3–8)	< 0.01^*^ < 0.01^*^	0.66
PICC (%) days, median (IQR)	70 (8.0) 4 (3–6.75)	292 (14.0) 6 (3.75–9)	< 0.01^*^ < 0.01^*^	0.85

*OR* Odds ratio, *ICU* Intensive care unit, *PICU* Paediatric intensive care unit, *EMS* Emergency medical service, *IMV* Invasive mechanical ventilation, *IQR* Interquartile range, *NPPV* Non-invasive mechanical ventilation, *CHDF* Continuous hemodiafiltration, *PEX* Plasma exchange, *PMX-DHP* Polymyxin B immobilized fiber column direct hemoperfusion, *ECMO* Extracorporeal membrane oxygenation, *ICP* Intracranial pressure, *CVC* Central venous catheter, *A-line* Arterial line, *PICC* Peripheral inserted central catheter

*p <  0.01

Table 5 shows the patient’s characteristics divided by the four age categories. There were significant differences of PIM2, chronic conditions and transport distance, between two group in all of the four age categories, except for adolescent. Mortality was significant larger in EMS group than family transport group only in the school age category (24 patients, 5.1% vs.0 patients, 0%; *p* <  0.01).

**Table 5:** Patient’s characteristics across the four age categories

Age category	Infant			Toddler			School age			Adolescent		
	Family transport group (*n* = 278)	EMS transport group (*n* = 606)	*p*-values	Family transport group (*n* = 352)	EMS transport group (*n* = 878)	*p*-values	Family transport group (*n* = 188)	EMS transport group (*n* = 468)	*p*-values	Family transport group (*n* = 53)	EMS transport group (*n* = 140)	*p*-values
PIM 2 (IQR)	1.3 (0.8–2.7)	1.9 (1.1–6.8)	< 0.01^*^	1.0 (0.7–2.8)	1.4 (1.0–4.9)	< 0.01^*^	1.0 (0.8–1.6)	1.2 (0.9–4.3)	< 0.01^*^	0.9 (0.7–1.5)	1.1 (0.9–4.7)	< 0.01^*^
Chronic conditions (%)	119 (42.8)	149 (24.6)	< 0.01^*^	252 (71.6)	401 (45.7)	< 0.01^*^	139 (73.9)	211 (45.1)	< 0.01^*^	41 (77.4)	84 (60.0)	0.028
Distance (km) (IQR)	5.2 (2.7–8.2)	5.5 (0.5–10)	< 0.01^*^	5.3 (3.3–9.6)	9.6 (4.9–16.8)	< 0.01^*^	5.0 (3.0–8.7)	9.7 (4.6–20)	< 0.01^*^	9.4 (4.5–16.9)	9.5 (4.5–16.9)	0.295
Mortality (%)	4 (1.4)	30 (5.5)	0.013	5 (1.4)	19 (2.2)	0.498	0	24 (5.1)	< 0.01^*^	0	7 (5.0)	0.193

*EMS* Emergency medical service, *PIM2* Pediatric index of mortality 2, *IQR* Interquartile range

*p <  0.01

Table 6 lists the patient’s characteristics divide by the PIM2 risk intervals. In all the risk intervals, number of patients with chronic condition was significant larger in the family transport group than EMS group. Transport distance was significant longer in EMS group than family transport group in all risk intervals, except for the high PIM2 risk category.

**Table 6:** Patient’s characteristics across the five risk intervals

PIM2 risk	< 1%			1–5%			5–15%			15–30%			> 30%		
	Family transport group (*n* = 344)	EMS transport group (*n* = 511)	*p*-values	Family transport group (*n* = 391)	EMS transport group (*n* = 1010)	*p*-values	Family transport group (*n* = 89)	EMS transport group (*n* = 312)	*p*-values	Family transport group (*n* = 24)	EMS transport group (*n* = 87)	*p*-values	Family transport group (*n* = 13)	EMS transport group (*n* = 146)	*p*-values
Chronic conditions (%)	201 (58.4)	191 (37.4)	< 0.01^*^	244 (62.4)	383 (37.9)	< 0.01^*^	64 (71.9)	149 (47.8)	< 0.01^*^	20 (83.3)	36 (41.4)	< 0.01^*^	12 (92.3)	63 (43.2)	< 0.01^*^
Distance (km) (IQR)	4.7 (2.9–8.4)	9.5 (4.3–16)	< 0.01^*^	5.7 (3.3-9.8)	9.6 (4.8-15)	< 0.01^*^	4.5 (2.7-8.5)	12 (6.4-25.8)	<0.01^*^	7.0 (3.7–21.7)	15.5 (10.1–46.0)	< 0.01^*^	9.5 (4.8–17.3)	10.4 (4.9–17.3)	0.507
Mortality (%)	2 (0.6)	1 (0.2)	0.568	2 (0.5)	4 (0.4)	0.674	1 (1.1)	4 (1.3)	1	1 (4.2)	5 (5.7)	1	3 (23.1)	65 (44.5)	0.156

*PIM2* Pediatric index of mortality 2, *EMS* Emergency medical service, *IQR* Interquartile range

*p < 0.01

The results of multivariable logistic analysis for survival outcome were shown in Table 7. There was no significant difference in survival rate between EMS and family transport group, matched by PIM2, chronic condition status and transport distance (OR:1.17, 95%CI:0.39–3.47, *p* = 0.78).

**Table 7:** Logistic regression analysis

	Survival OR	95% CI	*p*-value
Survival to hospital discharge (unmatched)	4.54	2.08 to 9.89	< 0.01^*^
Survival to hospital discharge (matched)	1.17	0.39 to 3.47	0.78

*OR* Odds ratio, *CI* Confidence interval

*P* < 0.01^*^

## Discussion

To the best of our knowledge, this is the first study that classified the patterns in ICU or PICU admissions based on their transportation method by analysing the JaRPAC database. Here, 47% of the patients were admitted to the ICU or PICU from the emergency department. We found that 29% of these patients were transported by their family and not EMS despite their severe condition that required PICU or ICU admission.

EMS for children has many components, which includes the parents, primary care providers, pre-hospital transport systems, emergency department services, and critical care services. Paediatric pre-hospital transport, an integral part of this system, has gradually developed over the past two decades and has received increasing attention in the paediatric literature [8, 13, 14]. Although the pre-hospital transport system has progressed in some countries, this system in Japan requires further attention. EMS personnel can only provide oxygen, ventilatory support with bag-bulb mask, or chest compression for paediatric patients; therefore, the system of pre-hospital care by emergency physicians is maintained in some areas [15]. According to a previous report, the need for paediatric advanced life support for the patient is limited, and it appears that family members do not hesitate in taking the patients to the hospital. In addition, the distance covered by the family while transporting the patient was significantly shorter than that of the EMS group. Transport distance was significant shorter in family transport group than EMS group in almost all of the age category, even though PIM2 risk was high. It means that it did not take much time for the family member to drive the patient to the hospital due to easy access to the hospital, even though EMS was available for every patient without any difficulty.

In this study, 92.4% of the patients who were transported by their family were admitted because of intrinsic diseases, whereas 23.4% of those transported by the EMS were brought because of extrinsic diseases, in particular, significantly higher cases of traffic accidents (76.3%). Chen et al. reported that a higher number of trauma patients require oxygenation or fluid infusion; therefore, it is reasonable that more trauma patients were transported by EMS [16]. By contrast, 60% of the patients transported by their family had certain chronic conditions, and their families would regularly take their children to the hospital; therefore, it is not a difficult decision for families to drive their children to the hospital on their own.

The reasons for admission differed between the two groups. In the family transport group, a significantly major reason for admission to the ICU or PICU was respiratory failure, among the intrinsic diseases. In the EMS group, the major reason for admission was neuromuscular disease, such as a seizure. More patients with seizures were transported by EMS due to the need for rapid resuscitation. Haque et al. reported that more than half of the patients were admitted to the ICU or PICU from the emergency department because of neuromuscular disease or respiratory failure [1]. In the present study, a similar trend was observed. PIM2 was higher in the EMS group than in the family transport group, and a higher number of patients required mechanical ventilation, CV, A-line, CHDF, and ECMO; therefore, mortality and severity were significantly higher in the EMS group than in the family transport group. It is inferred that PIM2 scores were lower in the family transport group than in the EMS group; as a result, family members thought that it was better if they drove the patients to the hospital on their own. PIM2 scores were lower in the family transport group than in the EMS group, even though divide into age categories. This study also revealed that as PIM2 risk category increases, more patients were transported by EMS. Even though it was analysed by multivariable logistic analysis matched with PIM2, chronic condition status and transport, transport method was not affected survival rate of patients. Previous reports on adult patients with trauma or myocardial infarction also showed no significant difference in survival rate regardless of transport mode [17, 18].

Despite the relatively large sample size, this study has several limitations. First, a retrospective analysis was performed; therefore, associations only among the available data could be described. Second, although the JaRPAC database is the largest available database for critically ill paediatric patients, it does not capture all PICUs and ICUs in Japan; therefore, there may be a selection bias towards more academically focused or resource-rich PICUs and ICUs that were able to join JaRPAC. Moreover, this registry did not provide institutional characteristics or therapeutic levels. Third, the JaRPAC registry is based on PICU or ICU settings; hence, data on hospital mortality or long-term follow-up were unavailable. We did not control for variations in the specific types of therapy administered in the emergency department or transportation prior to admission. Fourth, there were no data regarding why EMS was not used; therefore, the reason the patient was driven by their family member to the hospital was not clear. Fifth, the number of outcomes, as mortality are smaller in family transport group than that of EMS transport group. There may be a selection in the classification of the group. Finally, there were no data relevant to the indications or timing of hospital visits.

## Conclusions

This study used a large-scale registry of critically ill paediatric patients in Japan to describe the transportation patterns of patients prior to admission to the PICU or ICU. It was also revealed that as PIM2 risk category increases, more patients were transported by EMS, but transport method did not affect the survival rate of the patients. The rate of patients with chronic conditions or those admitted due to respiratory failure was higher in patients transported by their family. Patients with chronic conditions and respiratory symptoms are more likely to be admitted to the ICU or PICU, so it is important to provide prompt respiratory care or medical interventions early on. Further prospective studies are needed to reveal the decision factors about the transportation methods of patients admitted to PICUs or ICUs.

## Data Availability

The datasets generated during and/or analysed during the current study are not publicly available due to including of privacy but are available from the corresponding author on reasonable request.

## Abbreviations

A-line: Arterial line catheterisation
CHDF: Continuous hemodiafiltration
CV: Central venous access catheterisation
ECMO: Extracorporeal membrane oxygenation
EMS: Emergency medical services
ICU: Intensive care unit
IMV: Invasive mechanical ventilation
JaRPAC: Japanese Registry of Pediatric Acute Care
PICC: Peripherally inserted central catheterisation
PICU: Paediatric intensive care unit
PIM2: Paediatric Index of Mortality 2
